# Effect of EGCG–Methacrylate-Functionalized Resin Infiltrant on White Spot Lesions: An In Vitro Study

**DOI:** 10.3390/jfb16010006

**Published:** 2024-12-29

**Authors:** Karin Landmayer, Bruna de Oliveira Iatarola, Talita Portela Pereira, Raquel Shimizu Mori, Alyssa Teixeira Obeid, Mariele Vertuan, Daniela Alvim Chrisostomo, Ana Carolina Magalhães, Lulwah Alreshaid, Paulo Henrique dos Santos, Anuradha Prakki, Luciana Fávaro Francisconi-dos-Rios

**Affiliations:** 1 Restorative Discipline, Dental Research Institute, Faculty of Dentistry, University of Toronto, 101 Elm Street, Toronto, ON M5G 1G6, Canada; karin.landmayer@gmail.com (K.L.);; 2 Department of Operative Dentistry, School of Dentistry, University of São Paulo, Avenida Professor Lineu Prestes, 2227, São Paulo 05508-000, SP, Brazil; 3 Department of Operative Dentistry, Endodontics and Dental Materials, Bauru School of Dentistry, University of São Paulo, Alameda Octávio Pinheiro Brisolla, 9-75, Bauru 17012-901, SP, Brazil; 4 Department of Biological Sciences, Bauru School of Dentistry, University of São Paulo, Alameda Octávio Pinheiro Brisolla, 9-75, Bauru 17012-901, SP, Brazil; 5 Department of Restorative and Prosthetic Dental Sciences, College of Dentistry, King Saud bin Abdulaziz University for Health Sciences, P.O. Box 3660, Riyadh 11481 K.S.A, Saudi Arabia

**Keywords:** antimicrobial agents, confocal laser scanning microscopy, dental white spots, catechin, icon infiltrant, ultraviolet spectrophotometry

## Abstract

This study evaluated the color change (ΔE_00_) and penetration depth (PD) of white spot lesions (WSLs) infiltrated with the resin infiltrant (Icon^®^) functionalized with methacrylate epigallocatechin-3-gallate (EGCG). To introduce polymerizable double bonds, EGCG was reacted with methacryloyl chloride (EM). Subsequently, the Icon resin infiltrant (I) was loaded with neat EGCG (IE) or EGCG–methacrylate (IEM) at 2 wt% each. WSLs were created on bovine enamel blocks and treated with I, IE, or IEM. Sound and untreated enamel surfaces were used as controls (C). Infiltrant PD (%) was determined by Confocal Laser Scanning Microscopy (CLSM, n = 12) analysis. For color change (ΔE_00_) determination (n = 14), ΔL, Δa, and Δb, half of each sample was kept sound as a reference area. The color was determined with a spectrophotometer. Data were statistically evaluated (*p* = 0.05). Surface morphology was obtained as a qualitative response variable using 3D CLSM. PD (%) did not differ statistically for I, IE, and IEM (*p* = 0.780). Groups I and IEM showed similar performance on color change (ΔE_00_) compared to the control group, while IE exhibited intermediate results, with no significant difference observed between the untreated, I, and IEM groups (*p* < 0.001). IEM promoted the masking of the WSL color without interfering with the PD.

## 1. Introduction

Accumulation of bacterial biofilm on tooth structures (large mounds of complex and structured microbial communities) is considered one of the primary etiological factors of dental caries formation [1]. These microorganisms metabolize types of sugars, producing acids that diffuse into the enamel, thereby leading to a demineralization process when an imbalance in the pH occurs [1]. The process is initiated as a subsurface demineralization of enamel, with an increase in the porosity of the outer layer beneath the surface layer, which is more mineralized and not initially ruptured [1,2,3].

At this stage, the demineralized and non-cavitated dental structure is presented as a white spot lesion [3]. The body of the lesion, porous due to the dissolution of enamel prisms, is filled with water/saliva, which presents a refractive index (1.33) different from the surrounding sound enamel (1.62), resulting in a whitish appearance [1,4]. The contrast in optical properties becomes even more evident when the lesion is dried with an air stream (refractive index around 1.00), as a consequence of the replacement of the aqueous components within pores [1,4].

White spot lesions (WSLs) significantly compromise esthetic appearance, particularly when anterior teeth are affected [5], being readily discernible by the patient [6,7]. Moreover, white spot lesions jeopardize oral health, as they can progress and potentially lead to cavitation if left untreated [3]. This early stage in the caries process is crucial for diagnosis since the lesions can be reversed and halted through conservative or minimal intervention strategies [8].

A promising approach, both in terms of arresting the lesion and masking the white appearance, involves the infiltration of low-viscosity resin into the pores of the body of the lesion to fill the intracrystalline space [9,10,11,12]. The penetration occurs as a result of capillary forces, facilitated by the action of hydrochloric acid applied prior to the resin infiltrant, leading to a reduced contact angle with enamel and an increased surface tension [10,11,12,13,14,15].

Regarding esthetics, the resin infiltrant has the capability to mask the white spot, but some factors such as the depth and histopathological features of the lesion may influence its effectiveness [9]. The refractive index of the resin infiltrant (1.52) closely resembles that of hydroxyapatite (1.62) [16], thereby justifying the camouflage of the infiltrated lesion and its enhanced performance compared to non-intervention or other conservative or minimally invasive treatments [17,18]. Although the white appearance may not always entirely disappear, the size of the lesion significantly reduces [17], and the color difference (ΔE) between the infiltrated lesion and the adjacent sound enamel remains low, thus satisfying the esthetic desires of patients [9,19,20,21]. Recently, clinical studies have shown adequate longevity of the treatment and demonstrated color stability for a period of up to 8 years [22,23,24].

In addition, the resin infiltrant blocks the diffusion pathways of cariogenic acids, thus preventing the progression of the lesion [11,25,26,27]. Although the infiltrant surrounds the enamel prims forming a mechanical barrier, some remnants of the prims may remain exposed and uncoated [28]. Furthermore, the surface roughness of an infiltrated lesion is higher when compared to sound enamel [28], and bacterial adhesion forces are directly related to the greater roughness of a resinous material [29]. This surface texture seems to facilitate biofilm adhesion, especially in the cervical or interproximal areas of the teeth, which are susceptible to plaque stagnation [30].

Surface roughness becomes more pronounced over time as bacterial colonization accelerates material degradation, which in turn increases surface porosity [31,32]. Additionally, demineralized areas not reinforced by resin are more susceptible to biodegradation in an aqueous environment [28,33]. This susceptibility is facilitated by the hydrophilic nature of triethylene glycol dimethacrylate (TEGDMA), a component of the infiltrant [14,34,35]. The monomer TEGDMA has low viscosity and has been shown to present high-water sorption and polymerization shrinkage values, [13,36] with a high extent of oxygen inhibition after polymerization [37]. All these factors may influence the sealing capacity and longevity of the infiltrant [37], contributing to the formation of voids and increased surface roughness [38,39,40].

A strategy to minimize the effects of the acidic by-products of stagnant biofilm on infiltrated enamel might be related to the incorporation of antimicrobial agents into resin infiltrants. Catechins may be a compound of choice due to their binding capacity to bacterial membranes, thereby damaging bacterial membranes and hindering bacterial adhesion to the surface [41,42]. Such antimicrobial activity has high efficacy for epigallocatechin-3-gallate (EGCG), the most abundant catechin in green tea [41,43].

EGCG has been demonstrated to inhibit glucosyltransferase and alpha-amylase activities, enzymes responsible for synthesizing polysaccharides that initiate bacterial adhesion to the tooth, and for performing a crucial role in carbohydrate metabolism [42,44,45,46]. Hence, EGCG can inhibit the growth of cariogenic bacteria, as well as the production of acids, a property that is also attributed to its buffering capability [47].

In this manner, several studies have investigated the effect of EGCG passively incorporated into different copolymers containing TEGDMA. At concentrations of 2% or higher in weight, although it significantly reduces the survival of *Streptococcus mutans*, EGCG may also impair polymerization conversion [48,49]. As an alternative approach, the chemical modification of EGCG with methacrylic groups allows its incorporation into TEGDMA in larger quantities without interfering with the polymerization process [50]. Moreover, EGCG–methacrylate stabilizes the polymeric matrix by reducing the presence of -OH groups in the copolymers and through increased cross-linking [50].

Thus, this study aimed to evaluate the penetration depth of a commercial resin infiltrant (Icon^®^; DMG, Hamburg, Germany) when functionalized with EGCG–methacrylate into artificial white spot lesions. The ability of the novel material to minimize the color difference between the white lesion and the adjacent sound enamel was also evaluated. The null hypotheses are that (1) the incorporation of antimicrobial agent, either neat EGCG or EGCG–methacrylate, into the resin infiltrant will not alter the material depth of penetration into WSLs; (2) the incorporation of an antimicrobial agent, either neat EGCG or EGCG–methacrylate, into the resin infiltrant will not alter the color difference between treated WSLs and adjacent sound enamel.

## 2. Materials and Methods

### 2.1. Samples Preparation and Experimental Groups

This study was approved by the local Ethics Committee on Animals Use (protocol number 006/2023-I, with the approval granted on 5 May 2023), after which 150 bovine teeth were obtained at a slaughterhouse. The teeth were cleaned using periodontal curettes and stored in 0.1% thymol solution at 4 °C until use.

For penetration depth, the sample size was calculated (http://estatistica.bauru.usp.br/calculoamostral/, accessed on 14 May 2022) considering that the commercial resin infiltrant (Icon; DMG, Hamburg, Germany) penetrates 41.94 ± 5.73 µm into artificial WSLs [51]. An estimated standard deviation of 5.73, an effect size of 7 µm, and alpha and beta errors of 5 and 20%, respectively, were used. The calculation showed that 11 teeth per group were required; 12 samples were used. For color analysis, a ΔE_00_ value of 6.28 ± 0.531 was considered between the WLSs and the adjacent sound enamel, with the acceptability thresholds for CIEDE2000 set at 0.8. The sample size was calculated using an estimated standard deviation of 0.531 and an effect size of 0.8, along with alpha and beta errors of 5 and 20%. The calculation indicated that 14 samples per group were required.

Using an automatic cutting machine (Isomet Low-Speed Saw; Buehler Ltd., Lake Buff, IL, USA), specimens measuring 4 × 4 mm (n = 50) and 6 × 3 mm (n = 100) were obtained from the mid-coronal region of the teeth. The dentin was flattened in a polishing machine (EcoMet; Buehler Ltd., Lake Buff, IL, USA) with 1200-grit SiC paper, and the enamel was flattened and polished using a sequence of abrasive papers with decreasing grit sizes (400-, 800-, 1200-, 2400-, and 4000-grit), all under constant water cooling.

The selection of specimens for this study was based on surface microhardness values, which were tested using Knoop hardness tester (HMV-G21DT, Shimadzu Co., Tokyo, Japan) with a load of 50 g for 10 s. The microhardness mean value was determined for each specimen from five random indentations. Specimens that presented a standard deviation greater than 10% of their microhardness mean (intra-block variability) and individual microhardness mean higher or lower than 10% of the microhardness mean calculated for all blocks (inter-block variability) were excluded from this study. Also, the specimens that presented with microhardness lower than 300 KHN and higher than 350 KHN were excluded from this study. Selected specimens were randomly distributed to different experimental groups and for the WSL protocol validation.

### 2.2. Transverse Microradiography (TMR) for WSL Validation

**Figure 1:**
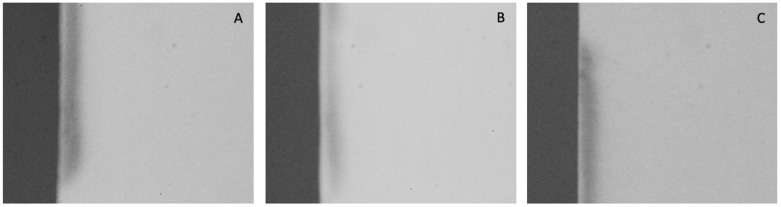
Representative transverse microradiography (TMR) images of enamel white spot lesion from sample 1 (**A**), sample 2 (**B**), and sample 3 (**C**). All samples exhibited an outer surface layer corresponding to the pseudo-intact surface layer over the body of lesion typical of caries white spot lesions.

Three specimens (6 × 3 mm) were designated for the protocol validation of WSL simulation, whereby a central window of 3 × 3 mm enamel area was preserved, with all other surfaces covered by two layers of nail varnish (Colorama; L’Oréal Brasil Comercial de Cosméticos Ltd., Rio de Janeiro, RJ, Brazil). In this central window, the WSL was induced by immersing samples in 32 mL of 50 mM acetate buffer solution without agitation at 37 °C for 64 h [51,52,53,54]. After 64 h, the specimens were sectioned into 3 slices, each with a demineralized area and two reference areas, and polished to 80–100 µm thickness. Afterward, the slices were submitted to microradiograph using an X-ray generator (Softex, Tokyo, Japan) on the glass plate at 20 kV and 20 mA (at a distance of 42 cm) for 13 min and analyzed by a transmitted light microscope with a 20× objective (Zeiss, Oberkochen, BW, Germany). The obtained images were evaluated using Inspektor Research System BV calculation software (version 2006) (Amsterdam, the Netherlands). Thus, lesion depth (LD, μm), integrated mineral loss (∆*Z*, vol%∙μm), and average mineral loss over the lesion depth (R, vol%) were measured. The acquisition data are shown in Table 1, and the representative images are shown in Figure 1.

As the pseudo-intact superficial layer was maintained after 64 h, the same fresh solution was used for the entire study. For the 4 × 4 mm samples, the lesion was created on the entire enamel surface, while for 6 × 3 mm samples, the WSL was created on half of the surface (3 × 3 mm), while the other half remained sound. All surfaces, except those to be exposed to the solution, were covered with nail varnish. Subsequently, samples were immersed in 32 mL of 50 mM acetate buffer solution without agitation at 37 °C for 64 h.

### 2.3. Incorporation of EGCG and EGCG–Methacrylate into Icon

The synthesis of EGCG–methacrylate was carried out following a previous method [50]. In this study, EGCG–methacrylate was used with 33% of hydroxyl functionalized (EM), which means that one-third of the -OH groups within the EGCG molecule were functionalized with methacryloyl ester to render photopolymerizable double bonds. Synthesized material was incorporated into the Icon resin infiltrant (IEM) at 2% (m/m). Neat EGCG (E) was also incorporated into the resin infiltrant (IE) at the same concentration.

### 2.4. Confocal Laser Scanning Microscopy (CLSM) Evaluation

The 4 × 4 mm specimens were used for penetration depth analysis. All sides of the 4 × 4 mm specimens were covered with nail varnish, leaving only the WSL exposed for treatment. The lesions were etched with 37% phosphoric acid for 30 s, followed by a 30 s rinse. Each sample was immersed in 0.5 mL of an ethanolic solution of 0.1% red fluorophore Rhodamine B isothiocyanate for 12 h at 37 °C. The surface was dried using an airflow for 10 s. Icon dry (99% ethanol) and the resin infiltrant were applied according to the experimental groups (I: Icon resin infiltrant; IE: Icon resin infiltrant loaded with neat EGCG; or IEM: Icon resin infiltrant loaded with EGCG–methacrylate). The application procedure was the same for all infiltrants: The material was applied using a microbrush and remained on the surface for 3 min, excess material was removed with a cotton pellet, and light-cured (Valo Grand Cordless, Ultradent Products, Inc., South Jordan, UT, USA) for 40 s. The infiltrant was reapplied, left for 1 min, the excess was removed, and light-cured for 40 s.

The samples were sectioned into 3 slices and immersed in a 30% hydrogen peroxide solution for 12 h at 37 °C to bleach the Rhodamine B dye that had not infiltrated. After this period, they were washed for 60 s and polished to a thickness of 0.5 mm using 1200- and 2400-grit SiC papers. Each slice was then immersed in a 50% ethanolic solution of 100 μM sodium fluorescein (NaFl; Sigma Aldrich, St. Louis, MI, USA) for 3 min to stain the porous part not infiltrated, followed by a 60 s wash.

Two slices per specimen were randomly selected and analyzed under a Confocal Laser Scanning Microscope (LSM800, Carl Zeiss, Oberkochen, Germany). Two-dimensional images (xy-axis) were obtained using a 63× objective with oil immersion in dual fluorescence mode transmission (Rhodamine B with a filter of 565 nm to 617 nm and fluorescein from 410 nm to 546 nm). The image resolution was 1192 × 1192 pixels.

The penetration depth of the resin infiltrant was measured at 5 different points per slice, totaling 10 readings per sample. Each measurement was 15 μm from each other, and the analysis was performed using software (ImageJ2, version 2.14.0/1.54f). The region filled by the infiltrant was defined as where Rhodamine B staining occurred, and the area with fluorescein was considered the non-infiltrated region. Next, a division of the Rhodamine by fluorescein values was performed to obtain a penetration percentage.

### 2.5. Color Analysis

The 6 × 3 mm specimens were used for color analysis. Each sample was divided in half, the 3 × 3 mm half without the lesion was used as a control reference for the other half, which had the lesion treated. The color difference (ΔE) analysis was always performed on halves corresponding to the same tooth.

The halves with the lesion were etched using 37% phosphoric acid for 30 s [51], washed using water air spray for 30 s, and dried applying air jets. Then, Icon dry (99% ethanol) was applied for 30 s and air-dried for 30 s. Subsequently, the resin infiltrants (I, IE, or IEM) were applied according to the experimental groups as previously described. Afterward, samples were polished. Additionally, there was an untreated WSL group (L) and a control group (C) in which the enamel remained sound.

The samples were stored in distilled water for 24 h at 37 °C, and three readings per specimen were conducted under a UV spectrophotometer (QT-AA3000, Qualistest, Lauderdale, FL, USA). Samples were analyzed over a white background and using the standard illuminant D65 and a 2-degree standard observer. The data were collected based on three-dimensional spectra according to L* (lightness) and a* (red–green axis) and b* (yellow–blue axis) coordinates. ΔE_00_ values (CIEDE2000), ΔL, Δa, and Δb, representing the difference between the baseline and experimental halves, were calculated as follows:

∆E00 = {[∆L′/(k_L_S_L_)]^2^ + [∆C′/(k_C_S_C_)]^2^ + [∆H′/(k_H_S_H_)]^2^ + RT [∆C′/(k_C_S_C_)] [∆H′/(k_H_S_H_)]}^1/2^

### 2.6. Surface Morphology Analysis

For each group, an additional tooth underwent the same treatment as described for color analysis. After 24 h in distilled water, the samples were analyzed under a 3D Confocal Laser Scanning Microscope (Model 3D VK, Keyence, Osaka, Japan). Images were captured at 20× magnification as the qualitative response variable, and the surface roughness was determined.

### 2.7. Statistical Analysis

The data were analyzed for normality (Shapiro–Wilk test) and homogeneity of variances (Levene’s test). The coordinate Δb was analyzed by one-way analysis of variance (ANOVA) and Tukey tests. For penetration depth, ΔE_00_, Δa, and ΔL values, non-parametric Kruskal–Wallis followed by Dwass–Steel–Critchlow–Fligner tests were used. The software used was Jamovi (version 2.3), and a significance level of 0.05 was adopted.

## 3. Results

### 3.1. Penetration Depth

**Figure 2:**
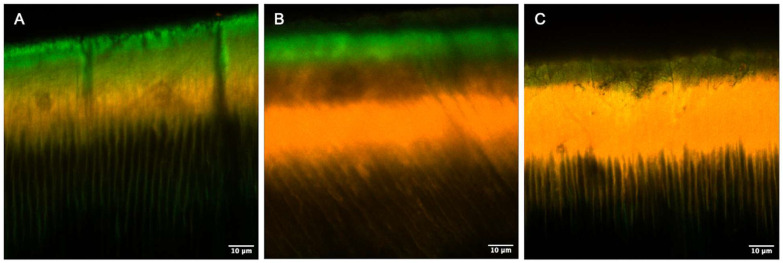
Illustrative images obtained by CLSM according to experimental groups, as follows: (**A**) resin infiltrant (I-Icon); (**B**) EGCG-functionalized Icon (IE); (**C**) EGCG–methacrylate-functionalized Icon (IEM). Rhodamine B dye (reddish areas) indicates the infiltrated region, while sodium fluorescein (greenish areas) highlights the non-infiltrated porous areas.

The ANOVA penetration depth analysis indicated that the resin infiltrant (Icon) functionalized with E or EM could infiltrate to the same depth as Icon (*p* = 0.780). The penetration depth was 95.2% for Icon, 94.2% for IE, and 95.2% for IEM. A representative image for each experimental group is shown in Figure 2.

### 3.2. Color

**Figure 3:**
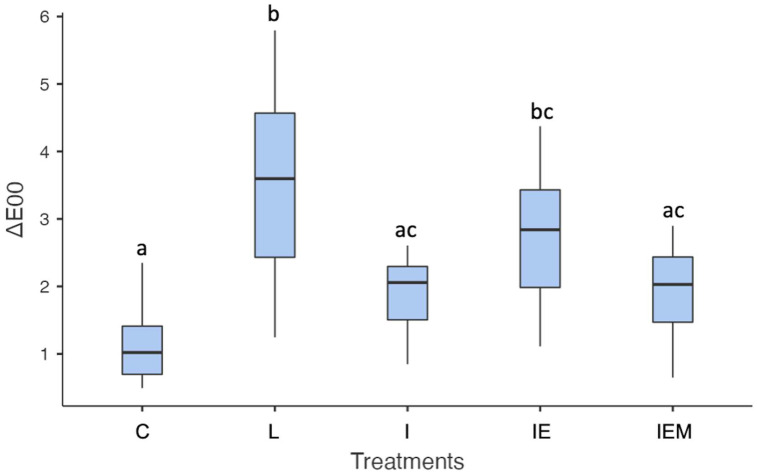
Data (25%/median/75%) of ΔE_00_ values of the control group (C), untreated white spot lesion (L), groups treated with Icon (I), epigallocatechin-3-gallate (EGCG)-functionalized Icon (IE), or EGCG–methacrylate-functionalized Icon (IEM). Different letters indicate statistically significant differences.

A significant difference was observed for ΔE_00_ values comparing the treatments (*p* < 0.001). Treatments involving Icon infiltrant (I) or Icon functionalized with EM (IEM) were effective in masking the white spot lesions, showing no significant difference compared to the control group (C, C vs. I *p* = 0.067; C vs. IEM *p* = 0.052). While the infiltrant modified with neat EGCG (IE) did not differ from the other treatments (I and IEM), it was statistically different from the control (C vs. IE *p* = 0.003; I vs. IE *p* = 0.095; IE vs. IEM *p* = 0.161, Figure 3).

**Figure 4:**
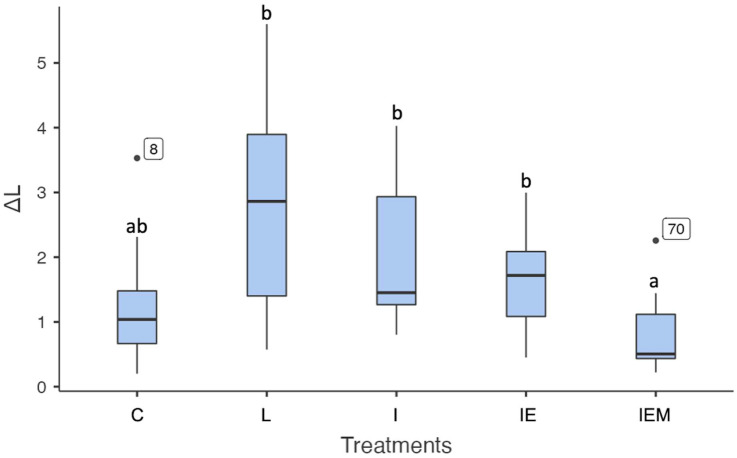
Data (25%/median/75%) of ΔL values of the control group (C), untreated white spot lesion (L), groups treated with Icon (I), EGCG-functionalized Icon (IE), or EGCG–methacrylate-functionalized Icon (IEM). Different letters indicate statistically significant differences.

Regarding lightness, no statistically significant differences were observed among all groups compared to the control group (C vs. L *p* = 0.075; C vs. I *p* = 0.234; C vs. IE *p* = 0.552; C vs. IEM *p* = 0.522). However, the group with EM incorporated into Icon (IEM) differed significantly from the white spot lesion treated with the Icon resin infiltrant (I), Icon resin infiltrant modified with E (IE), or untreated group (*p* < 0.001). Additionally, the Icon resin infiltrant (I) and resin infiltrant functionalized with E (IE) groups did not differ from the untreated group (L vs. I *p* = 0.829; L vs. IE *p* = 0.278, Figure 4).

**Figure 5:**
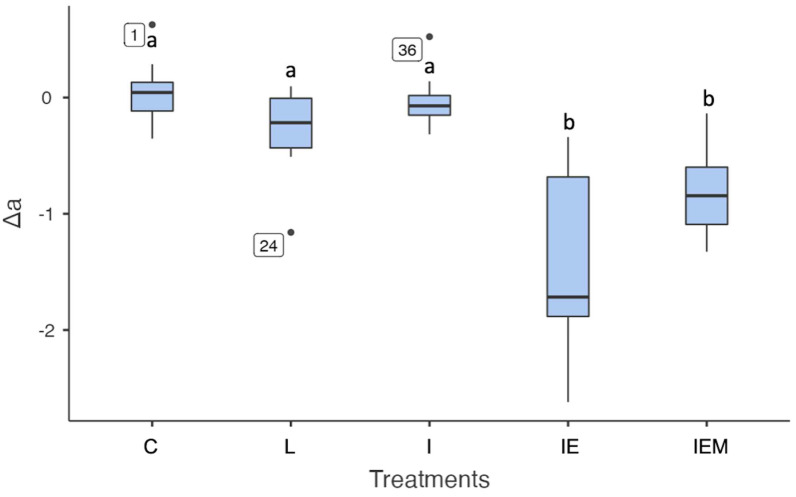
Data (25%/median/75%) of Δa values color coordinates (green–red axis) of the control group (C), untreated white spot lesion (L), groups treated with Icon (I), EGCG-functionalized Icon (IE), or EGCG–methacrylate-functionalized Icon (IEM). Different letters indicate statistically significant differences.

The specimens treated with Icon modified with E (IE) and EM (IEM) exhibited statistically significant differences in red–green coordinates compared to the other groups (*p* < 0.001). The untreated white spot lesion (L), the treatment with Icon resin infiltrant (I), and the control (C) groups had similar values (C vs. L *p* = 0.085; C vs. I *p* = 0.861; L vs. I *p* = 0.492, Figure 5).

**Figure 6:**
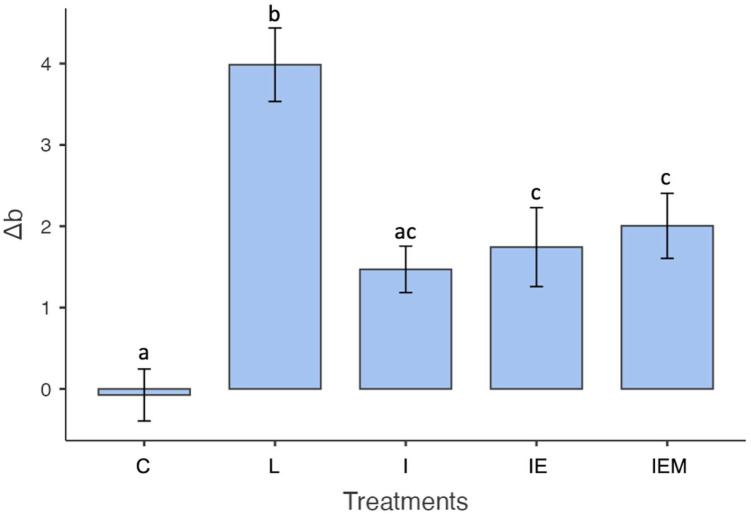
Means and standard deviations of Δb values color coordinate (blue–yellow axis) of the control group (C), untreated white spot lesion (L), groups treated with Icon (I), EGCG-functionalized Icon (IE), or EGCG–methacrylate-functionalized Icon (IEM). Different letters indicate statistically significant differences.

When comparing the experimental groups according to the b* coordinate, the yellow–blue axis significantly influenced the results (*p* < 0.001). The control group was statistically different from the untreated white spot lesion group (*p* < 0.001), while all treatments were similar to each other (I vs. IE *p* = 0.988; I vs. IEM *p* = 0.873; IE vs. IEM *p* = 0.990). Icon-functionalized groups (IE and IEM) differed from the control (C vs. IE *p* = 0.015; C vs. IEM *p* = 0.004) and untreated groups (L vs. IE *p* = 0.001; L vs. IEM *p* = 0.007), whereas only the I group showed no significant difference from the control (C vs. I *p* = 0.056, Figure 6).

### 3.3. Surface Morphology

**Figure 7:**
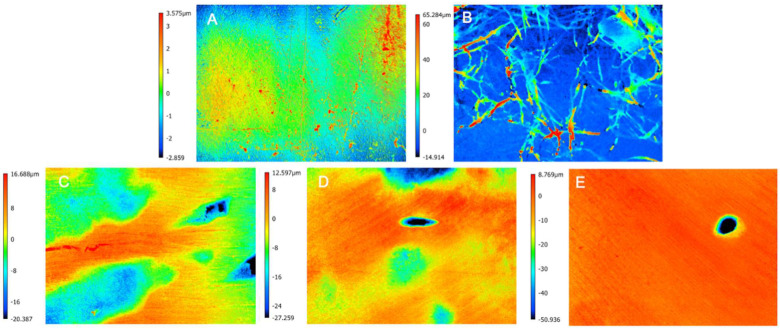
Surface morphology images obtained by CLSM according to experimental groups, as follows: (**A**) control (C), (**B**) untreated white spot lesion (L), (**C**) Icon resin infiltrant (I); (**D**) EGCG-functionalized Icon (IE); (**E**) EGCG-methacrylate-functionalized Icon (IEM).

As a qualitative variable, CLSM showed differences between the groups. The control group exhibited the smoothest and most uniform surface, while the surface of the untreated white spot lesion (WSL) was the most irregular. All treatments (I, IE, or IEM) resulted in a more uniform surface than the untreated surface, although they were less smooth than the control group. These findings are shown in Figure 7. The roughness values for the respective groups were as follows: control (C): 0.709, untreated white spot lesion (L): 7.780, Icon resin infiltrant (I): 4.703, Icon resin infiltrant modified with E (IE): 3.961, and Icon resin infiltrant modified with EM (IEM): 3.942.

## 4. Discussion

The commercially available resin infiltrant (Icon; DMG, Hamburg, Germany) has demonstrated efficacy in arresting the progression of incipient caries lesions while simultaneously masking their white appearance. This study confirms that even when white spot lesions were treated with the EGCG–methacrylate-modified infiltrant, the material penetrated into the demineralized enamel lesions and masked the whitish appearance by reducing the contrast between the white spots and the adjacent sound enamel.

This Icon resin infiltrant improves the surface roughness of the white spot lesion (WSL), though not to the level of sound enamel [55]. Similarly, this study observed through 3D confocal imaging that the surface of untreated WSLs exhibited greater irregularity, which was improved upon infiltration, irrespective of whether the Icon resin infiltrant was modified with EGCG or not. Nevertheless, the infiltrated area remains vulnerable to new cariogenic challenges, potentially leading to the development of new white spot lesions [56]. Additionally, after acid challenges, the enamel resulted in decreasing surface microhardness and increasing roughness [57,58], accompanied by numerous surface cracks [59]. These alterations in surface morphology facilitate biofilm accumulation.

To provide antibacterial effects and prevent caries recurrence, previous studies have incorporated quaternary ammonium monomers, nanosilver, and nanofibers of poli-lactic acid filled with calcium (SiO_2_-CaP) into an Icon resin infiltrant [60,61,62]. In general, the mechanical properties tested either improved or were not compromised by these additives [60,61,62]. However, even at low concentrations, the incorporation of silver into resin or ceramic caused darkening, and the color of the infiltrated WSLs on the anterior buccal surface significantly affects aesthetics [63,64]. Additionally, SiO_2_-CaP could alter the turbidity and opacity of the material [62]. Furthermore, the degree of conversion of the Icon resin infiltrant was influenced by the SiO_2_-CaP nanoparticles [62], potentially affecting the water sorption and solubility of the material, thereby diminishing its clinical longevity [34,62,65]. Also, there is a lack of evidence regarding whether the particles could impair the penetration depth of the Icon resin infiltrant [62].

This study used epigallocatechin gallate (EGCG) at a concentration of 2%. Despite being a natural crosslinker, it also demonstrates antibacterial, antioxidant, and anti-inflammatory effects that increase with higher concentration. However, when incorporated into TEGDMA (the main component of Icon resin infiltrant), neat EGCG at 2% concentration impaired the degree of conversion, gel content, and degree of swelling, which correlate with lower mechanical properties [50]. Therefore, 33% of EGCG –OH groups were replaced by methacrylic groups, increasing the number of polymerizable sites and copolymer crosslinking, thereby enhancing the mechanical properties, even at a concentration of 2%, that were previously compromised when incorporated into experimental resin infiltrant [50]. Also, covalently attaching EGCG to the resin matrix may prolong the antimicrobial effect.

The EGCG and EGCG–methacrylates incorporated into the Icon resin infiltrant can infiltrate the WSL in the same way as the non-modified material. Therefore, the first null hypothesis was not rejected. The incorporated particles could probably penetrate the pores created on the enamel with acid etching prior to infiltration. In this study, the diffusion pathways for infiltration were performed using phosphoric acid rather than hydrochloric acid, as recommended by the manufacturer. This choice was made since the artificial lesion used to standardize samples was shallow and had a thinner and less mineralized outer layer compared to a natural lesion [10]. Considering that the modified Icon is capable of infiltrating through the created porosities, further studies with deeper natural lesions are now encouraged.

The second null hypothesis was rejected. The color difference (ΔE_00_) between the WSL and adjacent sound enamel varied across treatments. The Icon resin infiltrant and EGCG–methacrylates within it masked the WSL, resulting in no significant difference between the treated and the reference area. On the other hand, neat EGCG incorporated into Icon (E) could not properly mask the whitish appearance; it was statistically similar to the untreated group. Although the E and EM groups had particles added at the same weight loading (2%), the molecular weight of EGCG (639.91) in functionalized copolymers is higher than that of neat EGCG (458.38). Thus, the quantity of EGCG particles present in Icon with E is higher; as for EM into Icon, there is also the weight of the methacrylic groups [50]. It is hypothesized that this is the reason why the color of the neat EGCG in the Icon group was unsatisfactory. Due to the presence of EGCG in the IE and IEM groups, there was a higher tendency towards red, which is natural given that EGCG is derived from green tea extract and has an orange–brown color. Nevertheless, this factor did not affect the ∆E00 for the EGCG–methacrylates group. Furthermore, the physical presentation of the E group as a white powder may have hindered the masking of the WSL, while for the EM group, it was pale brownish [50]. Another hypothesis that may explain the color difference of EGCG–methacrylate being statistically similar to unmodified Icon and different for EGCG is the likely copolymerization of EGCG–methacrylate with the Icon resin infiltrant, resulting in a more homogeneous surface.

Nevertheless, no treatment, including the Icon group (I), remained within the perceptibility and acceptability thresholds for ∆E_00_, which are 0.8 and 1.8, respectively [66]. Even the control group, which fell within the acceptability but not the perceptibility thresholds, suggests that the same tooth can exhibit color variations [67]. The cervical region typically has a more yellowish tone due to a greater amount of dentin and less enamel, while the incisal region is more translucent [67].

The values presented correspond to the CIEDE2000 formula, which offers a more precise reflection of color differences as perceived by the human eye, in contrast to the CIELab formula. CIEDE2000 provides better adjustments in color difference evaluation; therefore, it is preferred for clinical interpretation due to its enhanced accuracy in simulating human color perception [68].

A limitation of this study is the use of artificial bovine enamel lesions, which were not as deep as those found in natural teeth [10,69,70]. However, this was necessary for the standardization of this study, as it allowed for the polishing of a larger area, which is required for color evaluation. To confirm the creation of subsurface lesions, a gold-standard TMR test was used, ensuring precision and reproducibility.

Despite these limitations, the results obtained are promising. EGCG was added to the resin infiltrant to confer antibacterial properties to roughened areas, which are more susceptible to new cariogenic challenges [32,50]. To preserve the mechanical properties of the infiltrant without compromising its degree of conversion [50], EGCG–methacrylate was incorporated into the material, enabling penetration into the subsurface region while simultaneously masking the whitish appearance of white spot lesions.

Icon resin infiltrant modified with EGCG–methacrylate penetrates effectively and provides masking. It could clinically fulfill its original purposes without any loss of effectiveness and may contribute to caries disease control by acting on biofilm management [71]. This effect is particularly relevant in the immediate period following infiltration, when other non-invasive measures for disease control are implemented, especially for high-risk patients who typically present with active caries lesions [71].

## Figures and Tables

**Table 1:** Lesion depth (LD, μm), integrated mineral loss (∆*Z*, vol% μm), and average mineral loss over the lesion depth (R, vol%) for demineralized enamel samples.

	Sample	∆Z (vol%∙μm)	LD (μm)	R (vol%)
	1	2700	76.0	35.8
64 h	2	1610	66.1	24.3
	3	2645	85.7	31.6
		Average ∆Z (vol%.μm)	Average LD (μm)	Average R (vol%)
		2318.3	75.9	30.6

## Data Availability

The original contributions presented in the study are included in the article, further inquiries can be directed to the corresponding author.
